# The Woman at the Dig^1^

**DOI:** 10.3201/eid1011.AD1011

**Published:** 2004-11

**Authors:** Leo Dangel

**Keywords:** The Woman at the Dig, Leo Dangel, Poem, Poetry, archaeology

Tired from running a combine

all day through acres of wheat,

alone in front of the TV, I pay

attention because the show’s about

scientists digging up an ancient site.

I have no special interest in bones,

pottery, spearheads, or prehistoric

garbage dumps, and I always look past

the man describing animal migrations,

burial rites, or building design and try

to catch a glimpse of the women

working at the site − one of them

might be wearing cut-off jeans

and a halter top, clearing a patch

of ground with a trowel or brush.

These women are all experts.

You can tell by the way they look

at a bone chip or a pottery shard

they understand worlds about

the person who left it. Sifting soil,

they show more grace than contestants

in a Miss Universe pageant.

Years from now, when these farms

are ancient history, an expedition

with such a woman might come along.

I could drop something for her to find,

a pocketknife, a brass overalls button.

If only she could discover my bones.

My eyes would be long gone,

But I can see her form coming into focus

above me as she gently sweeps aside

the last particles of dust − her knee, thigh,

hip, shoulders, and finally, set off by sky

and spikes of sunlight, her face − a woman

who recognizes what she’s found.

Leo Dangel (b. 1941)

